# Crystal structure of 4-chloro-*N*-{[1-(4-chloro­benzo­yl)piperidin-4-yl]meth­yl}benzamide monohydrate

**DOI:** 10.1107/S1600536814018522

**Published:** 2014-09-03

**Authors:** K. Prathebha, D. Reuben Jonathan, S. Sathya, J. Jovita, G. Usha

**Affiliations:** a PG and Research Department of Physics, Queen Mary’s College, Chennai-4, Tamilnadu, India; b Department of Chemistry, Madras Christian College, Chennai-59, India

**Keywords:** crystal structure, piperidine, benzamide, hydrogen bonding

## Abstract

In the title compound, C_20_H_20_Cl_2_N_2_O_2_·H_2_O, the piperidine ring adopts a chair conformation with the two substituent benzene rings inclined to one another [dihedral angle 84.63 (9)°]. In the crystal, the components are linked by Ow—H⋯O, N—H⋯Ow (w = water) and C—H⋯O hydrogen bonds, generating a sheet structure lying parallel to (101).

## Related literature   

For the synthesis of the title compound, see: Prathebha *et al.* (2013 ▶). For the biological activity of piperdine derivatives, see: Parthiban *et al.* (2005 ▶, 2009 ▶, 2011 ▶). For related structures, see: Prathebha *et al.* (2013 ▶, 2014 ▶); Ávila *et al.* (2010 ▶).
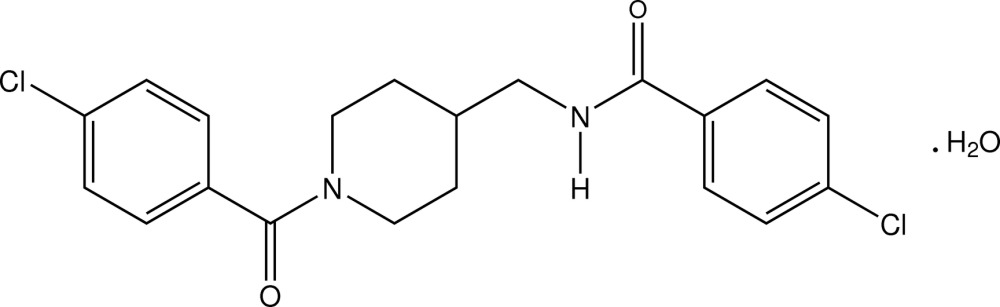


## Experimental   

### Crystal data  

C_20_H_20_Cl_2_N_2_O_2_·H_2_O
*M* *_r_* = 409.30
Monoclinic, 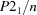

*a* = 8.965 (5) Å
*b* = 19.613 (5) Å
*c* = 11.456 (5) Å
β = 96.989 (5)°
*V* = 1999.3 (15) Å^3^
*Z* = 4
Mo *K*α radiation
μ = 0.35 mm^−1^
*T* = 293 K
0.20 × 0.15 × 0.10 mm

### Data collection  

Bruker APEII CCD diffractometer
Absorption correction: multi-scan (*SADABS*; Bruker, 2008 ▶) *T*_min_ = 0.939, *T*_max_ = 0.966
18886 measured reflections
4985 independent reflections
2967 reflections with *I* > 2σ(*I*)
*R*_int_ = 0.030

### Refinement  

*R*[*F* ^2^ > 2σ(*F* ^2^)] = 0.043
*wR*(*F* ^2^) = 0.120
*S* = 1.03
4985 reflections
252 parameters
2 restraints
H atoms treated by a mixture of independent and constrained refinement
Δρ_max_ = 0.23 e Å^−3^
Δρ_min_ = −0.30 e Å^−3^

### 

Data collection: *APEX2* (Bruker, 2008 ▶); cell refinement: *SAINT* (Bruker, 2008 ▶); data reduction: *SAINT*; program(s) used to solve structure: *SHELXS97* (Sheldrick, 2008 ▶); program(s) used to refine structure: *SHELXL97* (Sheldrick, 2008 ▶); molecular graphics: *ORTEP-3 for Windows* (Farrugia, 2012 ▶); software used to prepare material for publication: *SHELXL97* and *PLATON* (Spek, 2009 ▶).

## Supplementary Material

Crystal structure: contains datablock(s) I, New_Global_Publ_Block. DOI: 10.1107/S1600536814018522/zs2310sup1.cif

Structure factors: contains datablock(s) I. DOI: 10.1107/S1600536814018522/zs2310Isup2.hkl

Supporting information file. DOI: 10.1107/S1600536814018522/zs2310Isup3.cml

**Fig. 1.**
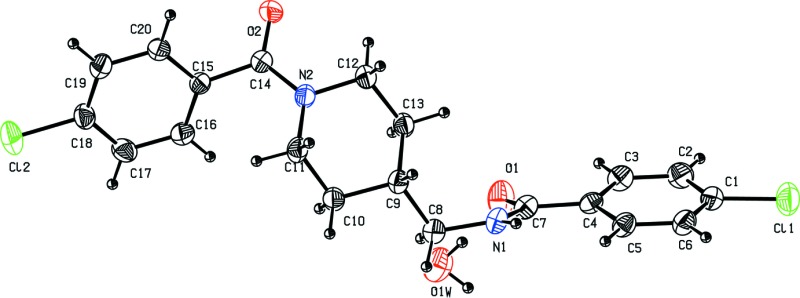
The molecular structure of the title compound, with displacement ellipsoids drawn at the 30% probability level.

**Fig. 2.**
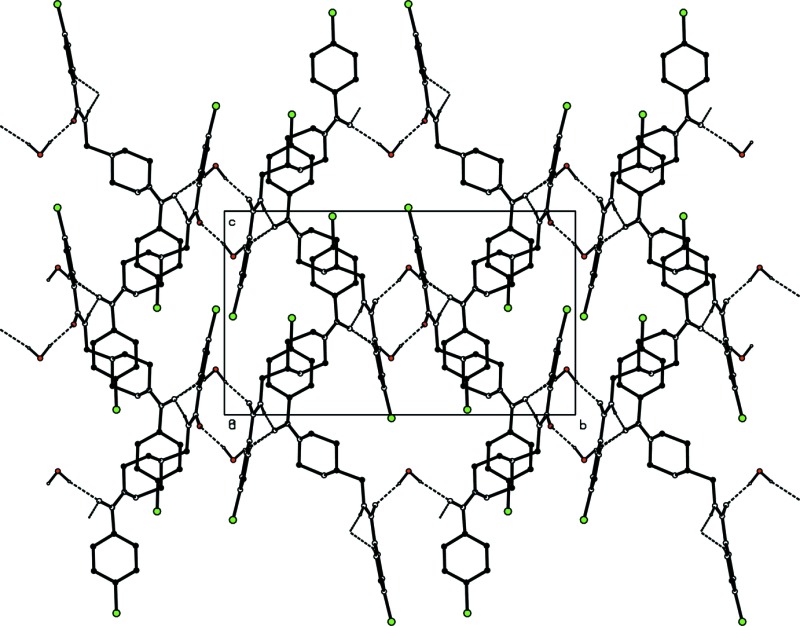
The packing of the molecules in the unit cell, viewed along a. Dashed lines indicate the hydrogen bonds.

. DOI: 10.1107/S1600536814018522/zs2310fig1.tif
The mol­ecular structure of the title compound, with displacement ellipsoids drawn at the 30% probability level.

a . DOI: 10.1107/S1600536814018522/zs2310fig2.tif
The packing of the mol­ecules in the unit cell, viewed along *a*. Dashed lines indicate the hydrogen bonds.

**Fig. 3.**
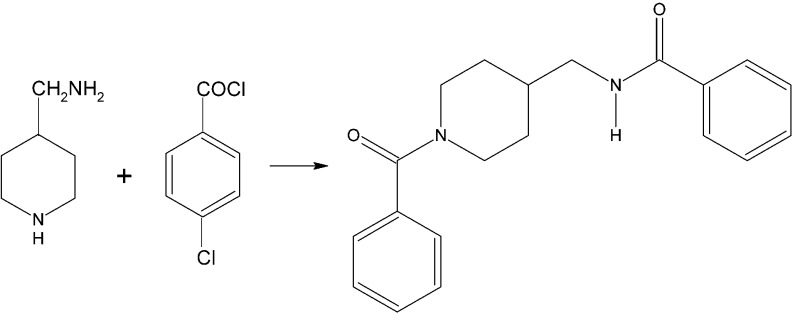
Experimental procedure

. DOI: 10.1107/S1600536814018522/zs2310fig3.tif
Experimental procedure

CCDC reference: 1014811

Additional supporting information:  crystallographic information; 3D view; checkCIF report

## Figures and Tables

**Table 1:** Hydrogen-bond geometry (Å, °)

*D*—H⋯*A*	*D*—H	H⋯*A*	*D*⋯*A*	*D*—H⋯*A*
N1—H1⋯O2^i^	0.86	2.19	3.043 (2)	171
O1*W*—H1*WA*⋯O2^ii^	0.87 (2)	2.11 (2)	2.972 (3)	169 (4)
O1*W*—H1*WB*⋯O1	0.87 (2)	1.96 (3)	2.802 (3)	163 (7)
C5—H5⋯O2^i^	0.93	2.36	3.212 (3)	152

Symmetry codes: (i) 
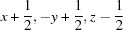
; (ii) 
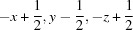
.

## supplementary crystallographic information

### S1. Comment

Piperidine and its derivatives have a high impact factor on the medical field due to their wide range of pharmacological activities. The piperidone molecule is also an important pharmacophore due to its broad-spectrum of biological actions ranging from anti-bacterial to anti-cancer (Parthiban *et al.*, 2005; 2009; 2011). The biological properties of these compounds are highly dependent on the type and location of substituents on the heterocyclic ring. We report in this communication, the synthesis and crystal structure of a new piperidine derivative, the title compound C_20_H_20_Cl_2_N_2_O_2_ . H_2_O.

In the title compound (Fig. 1), the bond lengths in the substituted benzene rings *A* and *B* are in good agreement with literature values. The piperidine ring is linked to these phenyl rings through a carbonyl group in a *trans* orientation. The C—N distances [1.335 (2)–1.464 (2)Å] are in the normal range and are in good agreement those in similar reported structures (Ávila *et al.*, 2010; Prathebha *et al.*, 2014). The bond angles around the N1 and N2 atoms [359.3 (4)° and 360.07 (1)°, respectively], show sp^3^ hybridization of the atoms. The presence of the carbonyl group linking the *A* and *B* rings contracts the bond angles C2—C1—C6 and C20—C15—C16 [118.17 (2) and 119.43 (2) Å, respectively] and expands the bond angles C3—C4—C5 and C17—C18—C19 [121.04 (2) Å and 121.51 (2) Å, respectively]. The bnzene rings *A* and *B* form dihedral angles of 53.9 (1)° and 43.7 (1)°, respectively, with the piperidine ring system. The piperidine ring (C13/C14/C15/C16/C17/N1) adopts a chair conformation with puckering parameters of *q*2 = 0.0184 (2) Å, φ2 = 35.47(6.16)°, *q*3 = 0.5583 (2) Å, *QT* = 0.5586 (2) Å and θ2 = 1.88 (2)°.

In the crystal, intermolecular water O—H···O hydrogen bonds form chains running along the *b* axis (Table 1). These chains are in turn linked by N—H···O and C—H···O hydrogen bonds giving a two-dimensional supramolecular layered structure lying parallel to (101) (Fig. 2).

### S2. Experimental

The procedure (Prathebha *et al.*, 2013; 2014) adopted in the synthesis of the typical diamide is re-presented here (Fig. 3). To 4-aminomethylpiperidine (0.02 mol) in a 250 mL round-bottomed flask, 120 mL of ethyl methyl ketone was added and stirred at room temperature. After 5 minutes, triethylamine (0.04 mol) was added and the mixture was stirred for 15 minutes. 4-Chlorobenzoyl chloride (0.04 mol) was then added and the reaction mixture was stirred at room temperature for about 2 h. A white precipitate of triethylammonium chloride was formed. It was filtered and the filtrate was evaporated to obtain the crude product which was recrystallized twice from ethyl methyl ketone: yield: 82%.

### S3. Refinement

H atoms were positioned geometrically and treated as riding on their parent atoms with C—H = 0.93–0.98 Å, N—H = 0.86 Å and O—H = 0.87 Å with *U*_iso_(H) = 1.5*U*_eq_(C-methyl) = 1.2*U*_eq_(N,C). The *U*_iso_ of the water H-atom were refined freely.

### Crystal data

**Table:**

C_20_H_20_Cl_2_N_2_O_2_·H_2_O	*Z* = 4
*M**_r_* = 409.30	*F*(000) = 856
Monoclinic, *P*2_1_/*n*	*D*_x_ = 1.360 Mg m^−^^3^
Hall symbol: -P 2yn	Mo *K*α radiation, λ = 0.71073 Å
*a* = 8.965 (5) Å	Cell parameters from 4985 reflections
*b* = 19.613 (5) Å	µ = 0.35 mm^−^^1^
*c* = 11.456 (5) Å	*T* = 293 K
β = 96.989 (5)°	Block, colourless
*V* = 1999.3 (15) Å^3^	0.20 × 0.15 × 0.10 mm

### Data collection

**Table:**

Bruker APEII CCD diffractometer	4985 independent reflections
Radiation source: fine-focus sealed tube	2967 reflections with *I* > 2σ(*I*)
Graphite monochromator	*R*_int_ = 0.030
ω and φ scan	θ_max_ = 28.3°, θ_min_ = 2.1°
Absorption correction: multi-scan (*SADABS*; Bruker, 2008)	*h* = −11→11
*T*_min_ = 0.939, *T*_max_ = 0.966	*k* = −26→26
18886 measured reflections	*l* = −15→15

### Refinement

**Table:**

Refinement on *F*^2^	Primary atom site location: structure-invariant direct methods
Least-squares matrix: full	Secondary atom site location: difference Fourier map
*R*[*F*^2^ > 2σ(*F*^2^)] = 0.043	Hydrogen site location: inferred from neighbouring sites
*wR*(*F*^2^) = 0.120	H atoms treated by a mixture of independent and constrained refinement
*S* = 1.03	*w* = 1/[σ^2^(*F*_o_^2^) + (0.0472*P*)^2^ + 0.401*P*] where *P* = (*F*_o_^2^ + 2*F*_c_^2^)/3
4985 reflections	(Δ/σ)_max_ < 0.001
252 parameters	Δρ_max_ = 0.23 e Å^−^^3^
2 restraints	Δρ_min_ = −0.30 e Å^−^^3^

### Special details

**Table:**

Geometry. All e.s.d.'s (except the e.s.d. in the dihedral angle between two l.s. planes) are estimated using the full covariance matrix. The cell e.s.d.'s are taken into account individually in the estimation of e.s.d.'s in distances, angles and torsion angles; correlations between e.s.d.'s in cell parameters are only used when they are defined by crystal symmetry. An approximate (isotropic) treatment of cell e.s.d.'s is used for estimating e.s.d.'s involving l.s. planes.
Refinement. Refinement of *F*^2^ against ALL reflections. The weighted *R*-factor *wR* and goodness of fit *S* are based on *F*^2^, conventional *R*-factors *R* are based on *F*, with *F* set to zero for negative *F*^2^. The threshold expression of *F*^2^ > σ(*F*^2^) is used only for calculating *R*-factors(gt) *etc*. and is not relevant to the choice of reflections for refinement. *R*-factors based on *F*^2^ are statistically about twice as large as those based on *F*, and *R*- factors based on ALL data will be even larger.

### Fractional atomic coordinates and isotropic or equivalent isotropic displacement parameters (Å^2^)

**Table:**

	*x*	*y*	*z*	*U*_iso_*/*U*_eq_	
C1	0.2856 (2)	0.04517 (10)	−0.36865 (17)	0.0557 (5)	
C2	0.1651 (2)	0.05009 (11)	−0.30553 (18)	0.0604 (5)	
H2	0.0676	0.0448	−0.3426	0.072*	
C3	0.1908 (2)	0.06295 (10)	−0.18659 (17)	0.0548 (5)	
H3	0.1096	0.0658	−0.1434	0.066*	
C4	0.33501 (19)	0.07168 (9)	−0.13004 (15)	0.0445 (4)	
C5	0.4537 (2)	0.06734 (10)	−0.19643 (16)	0.0508 (5)	
H5	0.5513	0.0734	−0.1601	0.061*	
C6	0.4299 (2)	0.05428 (11)	−0.31542 (17)	0.0550 (5)	
H6	0.5105	0.0517	−0.3591	0.066*	
C7	0.3542 (2)	0.08172 (10)	0.00066 (16)	0.0499 (5)	
C8	0.5234 (2)	0.10784 (10)	0.17983 (15)	0.0525 (5)	
H8A	0.6209	0.0872	0.2027	0.063*	
H8B	0.4497	0.0820	0.2167	0.063*	
C9	0.52633 (19)	0.18034 (10)	0.22638 (14)	0.0443 (4)	
H9	0.5999	0.2063	0.1877	0.053*	
C10	0.5769 (2)	0.18086 (10)	0.35899 (15)	0.0502 (5)	
H10A	0.5085	0.1533	0.3986	0.060*	
H10B	0.6764	0.1610	0.3745	0.060*	
C11	0.5798 (2)	0.25282 (11)	0.40699 (15)	0.0520 (5)	
H11A	0.6068	0.2517	0.4916	0.062*	
H11B	0.6553	0.2792	0.3731	0.062*	
C12	0.3794 (2)	0.28733 (11)	0.25369 (15)	0.0537 (5)	
H12A	0.4454	0.3159	0.2136	0.064*	
H12B	0.2794	0.3070	0.2418	0.064*	
C13	0.3756 (2)	0.21620 (10)	0.20237 (15)	0.0487 (4)	
H13A	0.3469	0.2189	0.1181	0.058*	
H13B	0.3000	0.1895	0.2357	0.058*	
C14	0.3605 (2)	0.32021 (10)	0.45609 (15)	0.0447 (4)	
C15	0.41215 (19)	0.31470 (9)	0.58496 (14)	0.0419 (4)	
C16	0.4207 (2)	0.25291 (10)	0.64373 (16)	0.0513 (5)	
H16	0.3991	0.2126	0.6023	0.062*	
C17	0.4615 (2)	0.25100 (11)	0.76433 (17)	0.0561 (5)	
H17	0.4668	0.2096	0.8043	0.067*	
C18	0.4939 (2)	0.31065 (11)	0.82394 (15)	0.0533 (5)	
C19	0.4847 (2)	0.37221 (11)	0.76756 (16)	0.0558 (5)	
H19	0.5066	0.4123	0.8095	0.067*	
C20	0.4425 (2)	0.37407 (10)	0.64751 (15)	0.0492 (4)	
H20	0.4345	0.4158	0.6086	0.059*	
N1	0.48785 (17)	0.10246 (8)	0.05253 (13)	0.0504 (4)	
H1	0.5560	0.1131	0.0090	0.061*	
N2	0.43298 (16)	0.28557 (8)	0.37952 (12)	0.0483 (4)	
O1	0.24827 (16)	0.07042 (10)	0.05634 (13)	0.0782 (5)	
O2	0.25144 (15)	0.35694 (7)	0.42328 (11)	0.0607 (4)	
O1W	0.1758 (2)	−0.02509 (12)	0.22242 (18)	0.0874 (5)	
Cl1	0.25541 (8)	0.02408 (4)	−0.51685 (5)	0.0906 (2)	
Cl2	0.54727 (9)	0.30789 (4)	0.97486 (4)	0.0908 (2)	
H1WA	0.188 (5)	−0.0575 (19)	0.172 (3)	0.19 (2)*	
H1WB	0.181 (9)	0.002 (4)	0.162 (5)	0.34 (4)*	

### Atomic displacement parameters (Å^2^)

**Table:**

	*U*^11^	*U*^22^	*U*^33^	*U*^12^	*U*^13^	*U*^23^
C1	0.0626 (12)	0.0528 (13)	0.0495 (10)	0.0066 (10)	−0.0011 (9)	−0.0074 (9)
C2	0.0482 (11)	0.0644 (14)	0.0657 (13)	0.0005 (10)	−0.0047 (9)	−0.0062 (10)
C3	0.0457 (10)	0.0572 (13)	0.0622 (12)	−0.0030 (9)	0.0088 (9)	−0.0050 (10)
C4	0.0466 (10)	0.0346 (10)	0.0528 (10)	−0.0020 (8)	0.0076 (8)	−0.0052 (8)
C5	0.0447 (10)	0.0519 (12)	0.0556 (11)	−0.0026 (9)	0.0056 (8)	−0.0118 (9)
C6	0.0534 (11)	0.0591 (13)	0.0539 (11)	0.0027 (9)	0.0119 (9)	−0.0091 (9)
C7	0.0498 (11)	0.0476 (12)	0.0538 (10)	−0.0021 (9)	0.0121 (9)	−0.0048 (9)
C8	0.0574 (11)	0.0545 (13)	0.0458 (10)	0.0015 (9)	0.0073 (8)	−0.0021 (9)
C9	0.0436 (9)	0.0514 (12)	0.0384 (8)	−0.0011 (8)	0.0074 (7)	−0.0037 (8)
C10	0.0437 (10)	0.0646 (14)	0.0420 (9)	0.0108 (9)	0.0035 (7)	−0.0027 (9)
C11	0.0414 (10)	0.0711 (14)	0.0427 (9)	0.0064 (9)	0.0013 (7)	−0.0124 (9)
C12	0.0588 (11)	0.0613 (13)	0.0396 (9)	0.0121 (10)	0.0000 (8)	−0.0028 (9)
C13	0.0477 (10)	0.0586 (12)	0.0386 (8)	0.0028 (9)	0.0001 (7)	−0.0037 (8)
C14	0.0462 (10)	0.0449 (11)	0.0431 (9)	0.0019 (9)	0.0064 (7)	−0.0014 (8)
C15	0.0413 (9)	0.0459 (11)	0.0392 (8)	0.0041 (8)	0.0083 (7)	−0.0010 (8)
C16	0.0591 (11)	0.0428 (11)	0.0520 (10)	0.0007 (9)	0.0068 (8)	−0.0011 (9)
C17	0.0653 (12)	0.0505 (13)	0.0532 (11)	0.0020 (10)	0.0098 (9)	0.0136 (10)
C18	0.0575 (11)	0.0655 (14)	0.0379 (9)	0.0043 (10)	0.0104 (8)	0.0034 (9)
C19	0.0689 (13)	0.0513 (13)	0.0472 (10)	0.0008 (10)	0.0072 (9)	−0.0079 (9)
C20	0.0597 (11)	0.0425 (11)	0.0459 (9)	0.0018 (9)	0.0080 (8)	0.0027 (8)
N1	0.0529 (9)	0.0543 (10)	0.0456 (8)	−0.0062 (8)	0.0124 (7)	−0.0097 (7)
N2	0.0454 (8)	0.0599 (10)	0.0389 (7)	0.0093 (7)	0.0016 (6)	−0.0078 (7)
O1	0.0602 (9)	0.1147 (14)	0.0636 (9)	−0.0184 (9)	0.0238 (7)	−0.0073 (9)
O2	0.0645 (8)	0.0697 (10)	0.0478 (7)	0.0263 (7)	0.0061 (6)	0.0034 (7)
O1W	0.0768 (11)	0.1049 (15)	0.0855 (12)	−0.0057 (10)	0.0296 (9)	−0.0120 (12)
Cl1	0.0863 (4)	0.1263 (6)	0.0551 (3)	0.0222 (4)	−0.0080 (3)	−0.0227 (3)
Cl2	0.1259 (6)	0.1059 (6)	0.0391 (3)	0.0055 (4)	0.0041 (3)	0.0075 (3)

### Geometric parameters (Å, º)

**Table:**

C1—C6	1.373 (3)	C11—H11A	0.9700
C1—C2	1.374 (3)	C11—H11B	0.9700
C1—Cl1	1.736 (2)	C12—N2	1.463 (2)
C2—C3	1.377 (3)	C12—C13	1.513 (3)
C2—H2	0.9300	C12—H12A	0.9700
C3—C4	1.385 (3)	C12—H12B	0.9700
C3—H3	0.9300	C13—H13A	0.9700
C4—C5	1.384 (3)	C13—H13B	0.9700
C4—C7	1.499 (2)	C14—O2	1.235 (2)
C5—C6	1.378 (3)	C14—N2	1.339 (2)
C5—H5	0.9300	C14—C15	1.496 (2)
C6—H6	0.9300	C15—C20	1.377 (3)
C7—O1	1.227 (2)	C15—C16	1.384 (3)
C7—N1	1.334 (2)	C16—C17	1.386 (3)
C8—N1	1.458 (2)	C16—H16	0.9300
C8—C9	1.518 (3)	C17—C18	1.368 (3)
C8—H8A	0.9700	C17—H17	0.9300
C8—H8B	0.9700	C18—C19	1.367 (3)
C9—C13	1.518 (3)	C18—Cl2	1.738 (2)
C9—C10	1.532 (2)	C19—C20	1.382 (2)
C9—H9	0.9800	C19—H19	0.9300
C10—C11	1.514 (3)	C20—H20	0.9300
C10—H10A	0.9700	N1—H1	0.8600
C10—H10B	0.9700	O1W—H1WA	0.872 (19)
C11—N2	1.464 (2)	O1W—H1WB	0.87 (2)
			
C6—C1—C2	121.02 (19)	C10—C11—H11B	109.5
C6—C1—Cl1	119.43 (16)	H11A—C11—H11B	108.1
C2—C1—Cl1	119.50 (16)	N2—C12—C13	110.51 (15)
C1—C2—C3	119.08 (18)	N2—C12—H12A	109.5
C1—C2—H2	120.5	C13—C12—H12A	109.5
C3—C2—H2	120.5	N2—C12—H12B	109.5
C2—C3—C4	121.31 (18)	C13—C12—H12B	109.5
C2—C3—H3	119.3	H12A—C12—H12B	108.1
C4—C3—H3	119.3	C12—C13—C9	112.27 (15)
C5—C4—C3	118.16 (17)	C12—C13—H13A	109.1
C5—C4—C7	123.68 (16)	C9—C13—H13A	109.1
C3—C4—C7	118.08 (16)	C12—C13—H13B	109.1
C6—C5—C4	121.20 (17)	C9—C13—H13B	109.1
C6—C5—H5	119.4	H13A—C13—H13B	107.9
C4—C5—H5	119.4	O2—C14—N2	121.73 (16)
C1—C6—C5	119.21 (18)	O2—C14—C15	118.73 (15)
C1—C6—H6	120.4	N2—C14—C15	119.53 (15)
C5—C6—H6	120.4	C20—C15—C16	119.44 (16)
O1—C7—N1	122.23 (18)	C20—C15—C14	118.05 (16)
O1—C7—C4	119.57 (17)	C16—C15—C14	122.37 (16)
N1—C7—C4	118.20 (16)	C15—C16—C17	120.06 (18)
N1—C8—C9	114.30 (15)	C15—C16—H16	120.0
N1—C8—H8A	108.7	C17—C16—H16	120.0
C9—C8—H8A	108.7	C18—C17—C16	119.27 (19)
N1—C8—H8B	108.7	C18—C17—H17	120.4
C9—C8—H8B	108.7	C16—C17—H17	120.4
H8A—C8—H8B	107.6	C17—C18—C19	121.51 (17)
C8—C9—C13	113.17 (15)	C17—C18—Cl2	119.09 (16)
C8—C9—C10	110.19 (15)	C19—C18—Cl2	119.40 (16)
C13—C9—C10	109.02 (14)	C18—C19—C20	119.14 (19)
C8—C9—H9	108.1	C18—C19—H19	120.4
C13—C9—H9	108.1	C20—C19—H19	120.4
C10—C9—H9	108.1	C15—C20—C19	120.57 (18)
C11—C10—C9	110.88 (16)	C15—C20—H20	119.7
C11—C10—H10A	109.5	C19—C20—H20	119.7
C9—C10—H10A	109.5	C7—N1—C8	122.88 (15)
C11—C10—H10B	109.5	C7—N1—H1	118.6
C9—C10—H10B	109.5	C8—N1—H1	118.6
H10A—C10—H10B	108.1	C14—N2—C11	125.27 (14)
N2—C11—C10	110.84 (15)	C14—N2—C12	120.41 (15)
N2—C11—H11A	109.5	C11—N2—C12	113.62 (14)
C10—C11—H11A	109.5	H1WA—O1W—H1WB	84 (5)
N2—C11—H11B	109.5		
			
C6—C1—C2—C3	−1.4 (3)	O2—C14—C15—C16	122.3 (2)
Cl1—C1—C2—C3	176.28 (17)	N2—C14—C15—C16	−57.7 (2)
C1—C2—C3—C4	0.7 (3)	C20—C15—C16—C17	−0.8 (3)
C2—C3—C4—C5	0.2 (3)	C14—C15—C16—C17	−176.49 (16)
C2—C3—C4—C7	−176.73 (19)	C15—C16—C17—C18	−0.4 (3)
C3—C4—C5—C6	−0.4 (3)	C16—C17—C18—C19	1.0 (3)
C7—C4—C5—C6	176.32 (18)	C16—C17—C18—Cl2	−179.19 (14)
C2—C1—C6—C5	1.2 (3)	C17—C18—C19—C20	−0.3 (3)
Cl1—C1—C6—C5	−176.49 (16)	Cl2—C18—C19—C20	179.87 (14)
C4—C5—C6—C1	−0.3 (3)	C16—C15—C20—C19	1.5 (3)
C5—C4—C7—O1	−164.2 (2)	C14—C15—C20—C19	177.37 (16)
C3—C4—C7—O1	12.5 (3)	C18—C19—C20—C15	−1.0 (3)
C5—C4—C7—N1	15.3 (3)	O1—C7—N1—C8	5.4 (3)
C3—C4—C7—N1	−167.93 (18)	C4—C7—N1—C8	−174.18 (16)
N1—C8—C9—C13	62.2 (2)	C9—C8—N1—C7	−104.7 (2)
N1—C8—C9—C10	−175.49 (14)	O2—C14—N2—C11	166.40 (19)
C8—C9—C10—C11	−179.77 (15)	C15—C14—N2—C11	−13.5 (3)
C13—C9—C10—C11	−55.0 (2)	O2—C14—N2—C12	−3.4 (3)
C9—C10—C11—N2	56.1 (2)	C15—C14—N2—C12	176.71 (17)
N2—C12—C13—C9	−54.7 (2)	C10—C11—N2—C14	132.70 (18)
C8—C9—C13—C12	177.72 (15)	C10—C11—N2—C12	−56.9 (2)
C10—C9—C13—C12	54.7 (2)	C13—C12—N2—C14	−133.45 (18)
O2—C14—C15—C20	−53.4 (2)	C13—C12—N2—C11	55.7 (2)
N2—C14—C15—C20	126.55 (19)		

### Hydrogen-bond geometry (Å, º)

**Table:**

*D*—H···*A*	*D*—H	H···*A*	*D*···*A*	*D*—H···*A*
N1—H1···O2^i^	0.86	2.19	3.043 (2)	171
O1*W*—H1*WA*···O2^ii^	0.87 (2)	2.11 (2)	2.972 (3)	169 (4)
O1*W*—H1*WB*···O1	0.87 (2)	1.96 (3)	2.802 (3)	163 (7)
C5—H5···O2^i^	0.93	2.36	3.212 (3)	152
C8—H8*B*···O1	0.97	2.43	2.789 (3)	102
C12—H12*B*···O2	0.97	2.34	2.738 (2)	104

Symmetry codes: (i) *x*+1/2, −*y*+1/2, *z*−1/2; (ii) −*x*+1/2, *y*−1/2, −*z*+1/2.
