# Supplementary figures and images for: Crystal structure of 4-chloro-N-{[1-(4-chloro­benzo­yl)piperidin-4-yl]meth­yl}benzamide monohydrate
Source: Acta Crystallogr Sect E Struct Rep Online. 2014 Sep 3;70(Pt 10):o1080. doi: 10.1107/S1600536814018522 (PMC4257193; doi:10.1107/S1600536814018522)

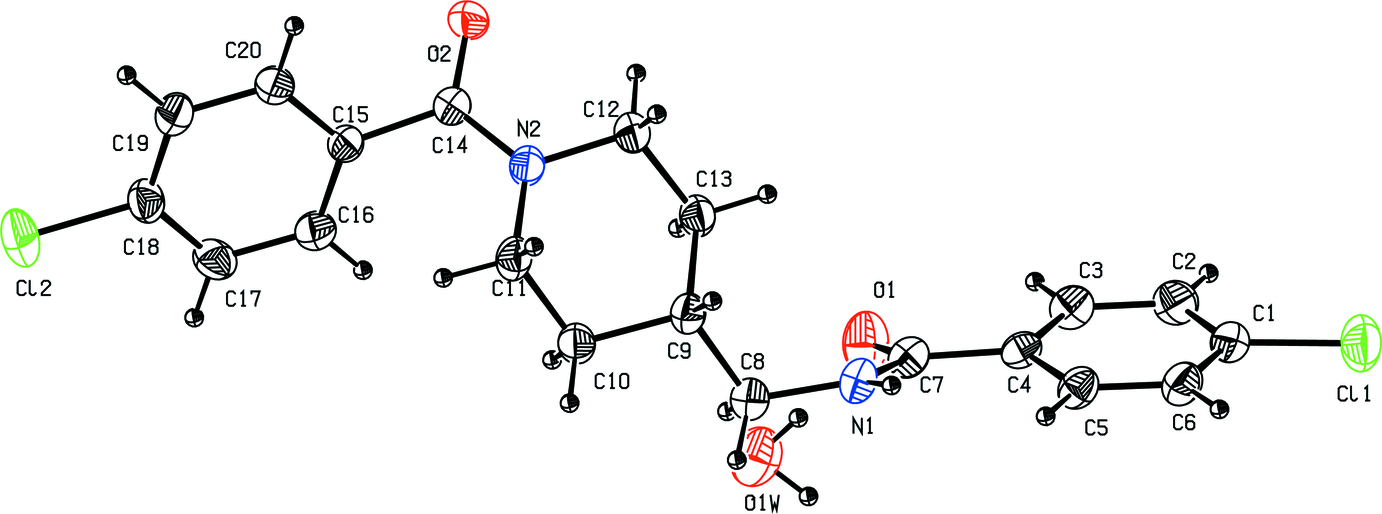

Supplement: Supplementary file 4 [file e-70-o1080-fig1.tif]

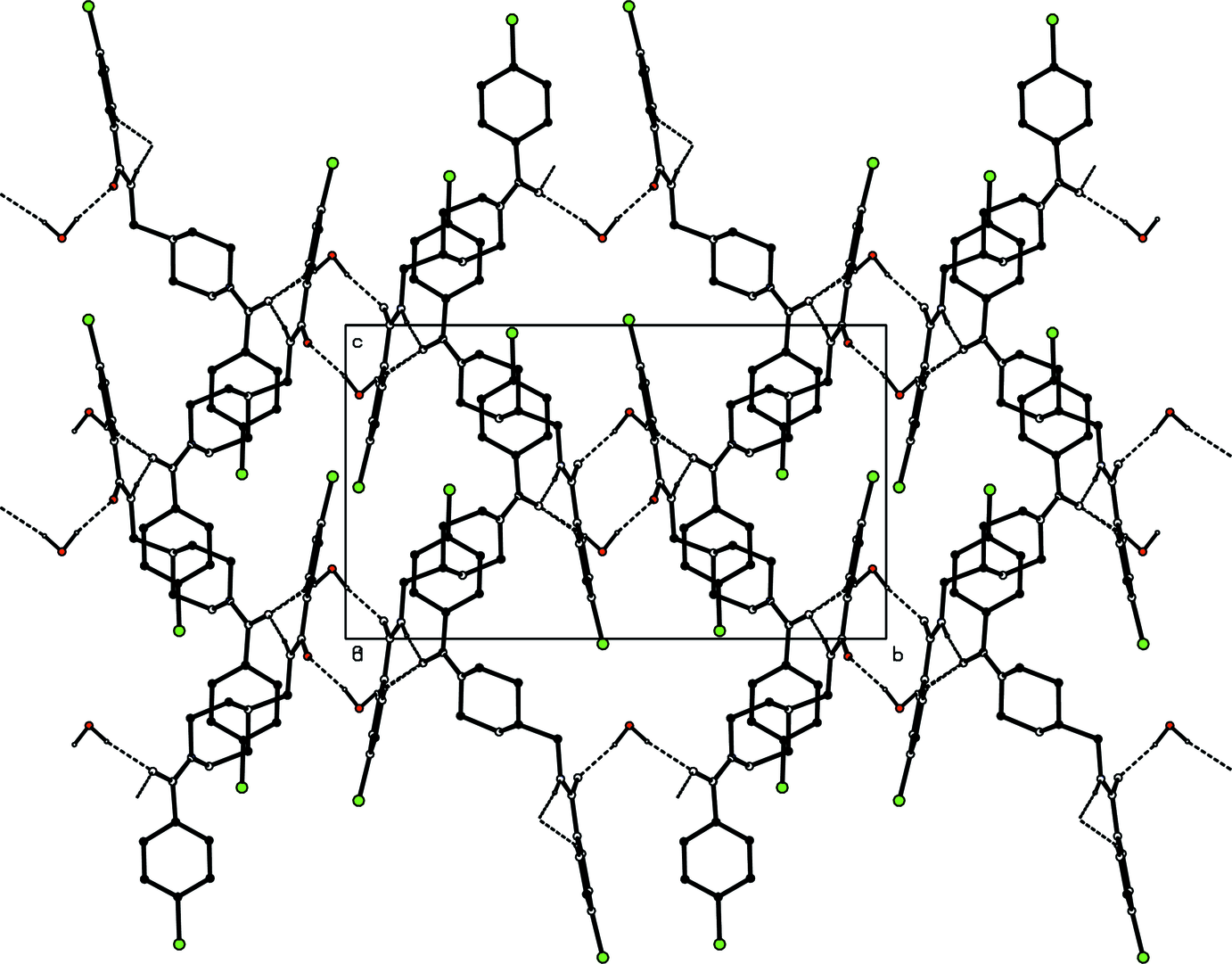

Supplement: Supplementary file 5 [file e-70-o1080-fig2.tif]
